# Surgical site infection: main cause of readmission of patients undergoing cardiac surgery

**DOI:** 10.1186/2047-2994-4-S1-P74

**Published:** 2015-06-16

**Authors:** BRN Barreiros, EF Bianchi, RTN Turrini, RA Lacerda, V De Brito Poveda

**Affiliations:** 1 Medical-Surgical, Nursing School of University of São Paulo, São Paulo, Brazil

## Introduction

Readmissions are a frequent problem in health institutions, bringing discomfort to the patient and family, and weigh the health system and society, therefore, cardiac patients suffer multiple readmissions to achieve full control of the disease or death, due to the complexity and the difficult management of cardiovascular diseases. Among the common causes of readmission there are the infections related to health care.

## Objectives

To characterize the readmission cardiac patients after the surgical procedure.

## Methods

This is a quantitative, retrospective, descriptive study, conducted through the medical records of patients who underwent cardiac surgery and subsequent readmission.

## Results

The sample consisted of 62 patients readmitted after performing heart surgery, these 66% were male, with predominant age group of 61 to 70 years and the comorbidities that stood out was hypertension (80%). The surgical site infection was the leading cause of rehospitalization, significantly associated to the variables obesity and dyslipidemia.

## Conclusion

The identification of patients at risk for the development of surgical site infection can minimize readmission rates and reduce the costs associated with care, and must therefore, be subject to a different action planning by the nursing staff.

## Disclosure of interest

None declared.

